# Chronic Temporal Lobe Epilepsy Is Associated with Enhanced Alzheimer-Like Neuropathology in 3×Tg-AD Mice

**DOI:** 10.1371/journal.pone.0048782

**Published:** 2012-11-14

**Authors:** Xiao-Xin Yan, Yan Cai, Jarod Shelton, Si-Hao Deng, Xue-Gang Luo, Salvatore Oddo, Frank M. LaFerla, Huaibin Cai, Gregory M. Rose, Peter R. Patrylo

**Affiliations:** 1 Department of Anatomy and Neurobiology, Central South University Xiangya School of Medicine, Changsha, Hunan, China; 2 Center for Integrated Research in Cognitive and Neural Sciences, Southern Illinois University Carbondale, Illinois, United States of America; 3 Department of Physiology, Southern Illinois University School of Medicine, Carbondale, Illinois, United States of America; 4 Department of Physiology and The Barshop Institute for Longevity and Aging Studies, University of Texas Health Science Center at San Antonio, San Antonio, Texas, United States of America; 5 Department of Neurobiology and Behavior, University of California Irvine, Irvine, California, United States of America; 6 Laboratory of Neurogenetics, National Institute on Aging, Bethesda, Maryland, United States of America; City of Hope National Medical Center and Beckman Research Institute, United States of America

## Abstract

The comorbidity between epilepsy and Alzheimer's disease (AD) is a topic of growing interest. Senile plaques and tauopathy are found in epileptic human temporal lobe structures, and individuals with AD have an increased incidence of spontaneous seizures. However, why and how epilepsy is associated with enhanced AD-like pathology remains unknown. We have recently shown β-secretase-1 (BACE1) elevation associated with aberrant limbic axonal sprouting in epileptic CD1 mice. Here we sought to explore whether BACE1 upregulation affected the development of Alzheimer-type neuropathology in mice expressing mutant human APP, presenilin and tau proteins, the triple transgenic model of AD (3×Tg-AD). 3×Tg-AD mice were treated with pilocarpine or saline (i.p.) at 6–8 months of age. Immunoreactivity (IR) for BACE1, β-amyloid (Aβ) and phosphorylated tau (p-tau) was subsequently examined at 9, 11 or 14 months of age. Recurrent convulsive seizures, as well as mossy fiber sprouting and neuronal death in the hippocampus and limbic cortex, were observed in all epileptic mice. Neuritic plaques composed of BACE1-labeled swollen/sprouting axons and extracellular AβIR were seen in the hippocampal formation, amygdala and piriform cortices of 9 month-old epileptic, but not control, 3×Tg-AD mice. Densities of plaque-associated BACE1 and AβIR were elevated in epileptic versus control mice at 11 and 14 months of age. p-Tau IR was increased in dentate granule cells and mossy fibers in epileptic mice relative to controls at all time points examined. Thus, pilocarpine-induced chronic epilepsy was associated with accelerated and enhanced neuritic plaque formation and altered intraneuronal p-tau expression in temporal lobe structures in 3×Tg-AD mice, with these pathologies occurring in regions showing neuronal death and axonal dystrophy.

## Introduction

Alzheimer's disease (AD) and chronic temporal lobe epilepsy (TLE) are classified as two distinct neurological disorders according to their major presenting symptoms. However, they share many pathological features including temporal lobe atrophy, neuronal death, gliosis, neuritic alterations and inflammation [1],[2],[3],[4],[5],[6],[7],[8],[9]. Further, temporal lobe hypometabolism is a premortem feature of AD, as it is for TLE during the interictal phase [10],[11],[12]. During an ictal discharge, increased glucose utilization occurs in the TLE focus exhibiting electroencephalographic spiking activity [12],[13].

Senile, or amyloid-containing, plaques were first described in epileptic human brain by Blocq and Marinesco in 1892 (detailed in [14]), prior to Alois Alzheimer's description of the first clinical case of AD in 1907 [15]. Much later, Mackenzie and Miller [16] reported senile plaques in approximately 10% of surgical temporal lobe samples from TLE cases that ranged in age from 36 to 61 years and did not exhibit dementia by standard neuropsychological tests. Furthermore, the age-related incidence of plaque formation in TLE patients was significantly increased relative to age matched non-epileptic controls. In addition to amyloid pathology, tauopathy or phosphorylated tau (p-tau) overexpression has been reported in epileptic human brain and in animal models of epilepsy [17],[18],[19],[20],[21].

Human and animal model data also link AD with an increased propensity for seizures or epileptiform neuronal activity [22],[23],[24],[25]. The incidence of seizures in individuals with AD-type dementia appears to be increased, especially in early-onset cases, although generalized convulsive episodes are rare [26]. In mouse models of AD the threshold for convulsant-induced seizures is lower, and spontaneous seizures have been observed [27]. Further, some AD mouse models exhibit increased neuropeptide Y immunoreactivity (IR) in hippocampal mossy fiber terminals [28], a change seen epileptic brain.

Despite these clinical and pathological commonalities between TLE and AD [23],[24], it remains unclear how and why epilepsy is associated with increased amyloid and tau pathology. We have shown early upregulation of the rate-limiting amyloidogenic enzyme β-secretase-1 (BACE1) inherent with axon terminal sprouting and dystrophy in transgenic AD mouse models, pointing to a leading role for pathological axonal sprouting in plaque development [29],[30],[31]. We have also characterized BACE1 elevation in temporal lobe structures in epileptic CD1 mice, which was associated with aberrant mossy fiber and neocortical axonal sprouting, but not with apparent extracellular amyloid deposition [32]. Thus, in the present study we used the triple transgenic mouse model of AD (3×Tg-AD) to explore the potential consequences of alterations in BACE1 expression on amyloid plaque formation, relative to tau pathology and neuronal death, following experimentally induced chronic epilepsy [33], [34],[35].

## Materials and Methods

### Ethics statement

Experimental use of mice in the present study was in accordance with the National Institutes of Health Guide for the Care and Use of Laboratory Animals. All procedures used were approved by the Animal Care and Use Committee of Southern Illinois University at Carbondale.

### Transgenic animals and induction of epilepsy

A colony of 3×Tg-AD mice was maintained at Southern Illinois University Carbondale, propagated from breeding pairs provided by Dr. Frank LaFerla [33]. Six- to 8-month old male transgenic mice were pre-treated with methylscopolamine (1.2 mg/kg, i.p.), followed 20 minutes later by pilocarpine (290–310 mg/kg, i.p.; both compounds were obtained from Sigma-Aldrich, St. Louis, MO) or vehicle (saline; i.p.). All animals were behaviorally monitored for acute seizures for the next 3–4 hours. Pilocarpine- but not saline-treated animals developed ≥3 convulsive seizures (class 3 seizure or greater) during the next several hours [35]. This degree of acute seizure severity has been shown to be sufficient to induce chronic epileptogenesis in mice [35],[36], and will be referred to as status epilepticus. Animals that survived the initial acute status (∼50% mortality occurred during the seizure induction period) were returned to their home cages in the animal facility and were video monitored for 8–10 hours per week to confirm the onset of spontaneous recurrent seizures following a post-status latent period. Epileptic 3×Tg-AD mice and age-matched saline treated controls were euthanized at 9 (n = 4 per group, seizures induced at 6 months old), 11 (n = 4 per group, seizures induced at 8 months old) and 14 (n = 4 per group, seizures induced at 8 months old) months of age. Because of the substantial mortality seen following pilocarpine treatment, four different sets of mice (vehicle or pilocarpine treated) were needed to accumulate the groups evaluated here. Some mice from each group contributed to the final sample for each population.

### Tissue preparation

Animals were overdosed with sodium pentobarbital (100 mg/kg, i.p.) and transcardially perfused with 0.01 M phosphate-buffered saline (PBS, pH 7.4), followed by 4% paraformaldehyde in PBS. Brains were removed, postfixed overnight in 4% paraformaldehyde, cryoprotected in 30% sucrose at 4°C, and then sectioned using a cryostat. Brains were individually labeled before sectioning by making small corner-cuts or needle punches in the cortex. This allowed for batch-processing of sections from different animals and subsequent identification of their sources during mounting and microscopic examination. For each brain, 12 sets of 30 µm-thick sections were consecutively collected in PBS in cell culture plates for immunohistochemistry using the avidin-biotin complex (ABC) method and Nissl stain. Additionally, twelve sets of 6 µm-thick sections were collected by thaw-mounting on positively charged microslides for double immunofluorescence studies. Sections from −2.0 to −4.0 mm caudal to bregma were used for densitometric analysis.

### Immunohistochemistry

Epileptic and control 3×Tg-AD mouse brains were processed under identical conditions. Sections were initially treated in 1% H_2_O_2_ in PBS for 30 minutes to block endogenous peroxidase, and then were pre-incubated in 5% normal horse serum with 0.1% Triton X-100 for 1 hour. For BACE1 labeling, sections were pretreated with 50% formamide and 50% saline sodium citrate buffer (2×SSC, pH 7.0) at 65°C for 60 minutes [30]. For Aβ antibody labeling, sections were first treated with 90% formic acid at room temperature for 20 minutes. Sections were thoroughly washed with PBS following these pretreatments, and then were incubated overnight at 4°C with primary antibodies (see Table 1) at pre-optimized concentrations in PBS containing the blocking serum. Sections were further reacted with biotinylated pan-specific secondary antibodies (horse anti-mouse, rabbit or goat IgG) at 1∶400 for 1 hour and then in freshly prepared avidin-biotin complex solution (1∶400; Vector Laboratories, Burlingame, CA, USA) for an additional hour. Immunoreactivity was visualized by developing the sections in a solution containing 0.05% diaminobenzidine (DAB) and 0.003% H_2_O_2_. Three 10-minute PBS washes were used between all incubations. To define nonspecific levels of immunolabeling in subsequent densitometric analysis, several brain-level-matched sections were processed in each experiment using the same immunohistochemical protocol described above except for the omission of the primary antibody.

**Table 1:** Primary antibodies used.

Antibody	Source	Product #	Dilution
mouse anti-Aβ1–16, 6E10	Signet	39320	(1∶4000)
mouse anti-Aβ1–42, 12F4	Signet	39240	(1∶2000)
rabbit anti-Aβ36–40	H. Mori	Ter40	(1∶2000)
rabbit anti-Aβ38–42	H. Mori	Ter42	(1∶2000)
rabbit anti-BACE1∀ (a.a. 46–163)	H. Cai	anti-BACE1∀	(1∶2000)
mouse anti-growth-associated protein	Sigma-Aldrich	G9264	(1∶4000)
mouse anti-polysialic acid neuronal cell adhesion molecule	Chemicon	MAB5324	(1∶4000)
mouse anti-microtubule associated protein-2	Sigma-Aldrich	M9942	(1∶2000)
rabbit anti-phosphor-Tau (*p*-Ser199/Ser202)	Sigma-Aldrich	T6819	(1∶3000)
mouse anti-phosphor-Tau (*p*-Ser396/Ser404)	P. Davies	PHF1	(1∶4000)
mouse anti-synaptophysin	Millipore	MAB329	(1∶4000)

For double immunofluorescence, sections were incubated in PBS containing 5% donkey serum and a pair of primary antibodies derived from different animal species (Table 1). Sections were then reacted for 2 hours with Alexa Fluor® 488 and Alexa Fluor® 594 conjugated donkey anti-mouse and rabbit IgGs (1∶200; Invitrogen, Carlsbad, CA, USA). Immunolabeled sections were counter-stained with bisbenzimide (Hoechst 33342, 1∶50000), washed in PBS and mounted using anti-fading medium.

Initial specificity tests for the primary antibodies entailed preabsorption of the primary antibody with neutralizing peptide and omission of the antibody in the incubation buffer. These controls yielded no specific labeling in brain sections. The specificity of the BACE1 antibody (anti-BACE1α) has been previously verified with western blot and immunohistochemical studies including using BACE1 knockout mice and wild type controls [30],[37],[38],[39].

### Imaging, densitometry, data analysis and statistical testing

Sections were examined using an Olympus (BX60) fluorescent microscope equipped with a digital camera and image analysis system (Optronics, Goleta, CA, USA). Images (1600×1200 pixels) were taken using 4× to 40× objective lens (10× ocular lens). Optical density (digital light unit per square mm, DLU/mm^2^) was measured in regions of interest using OptiQuant analysis software (Parkard Instruments, Meriden, CT, USA), with adjacent Nissl stain sections used for histological verification. Color images were first converted into black and white (grayscale) TIFF documents. Densitometry was then carried out using the OptiQuant software. For each brain, 3 equally-spaced coronal sections containing the amygdala (400 µm apart) were used for sampling of the dentate gyrus, amygdala and piriform cortex. Three other coronal sections (800 µm apart) were used for systematic densitometry of the hippocampal CA1 field. Specifically, the region of interest in CA1 consisted of a fan-shaped area demarcated by a dorsal and a ventral line. The former is a line perpendicular to the cortical surface that intersects with the merging point of the upper and low blades of the dentate granule cell layer. The ventral line was set at the level of the rhinal fissure and was also perpendicular to the cortical surface. Values of optical density (o.d.) were obtained from individual layers if the staining exhibited a differential laminar pattern (e.g., 6E10, p-tau, NeuN). Optical densities were also obtained from level-matched sections that were processed in the absence of a corresponding primary antibody, to determine the cut-off threshold values for calculating the specific o.d. in immunolabeled sections. Data were normalized to the means of controls in some cases (for graphical presentation), and statistically analyzed using one-way ANOVA with the Bonferroni post hoc test for multi-group comparisons, or Student's *t*-test for paired mean comparison (Prism GraphPad 4.1, San Diego, CA, USA). The minimal significance level was set at *p*<0.05.

## Results

We recently observed BACE1 elevation in association with mossy fiber sprouting, as well as axon terminal sprouting, in CA1, amygdala and entorhinal cortex, in epileptic CD1 mice. However, amyloid plaque pathogenesis was not observed in these non-transgenic rodents [32]. To determine whether TLE-induced BACE1 overexpression could promote amyloidogenic and p-tau pathology, we induced status epilepticus in 3×Tg-AD mice. In our colony of this transgenic strain, extracellular Aβ deposition in the forebrain routinely begins at 12–14 months of age [29]. Here we induced temporal lobe epilepsy in 6–8 month old 3×Tg-AD mice and examined the effects at 9, 11 or 14 months of age.

### Increased BACE1 and extracellular Aβ labeling in epileptic 3×Tg-AD mice

**Figure 1:**
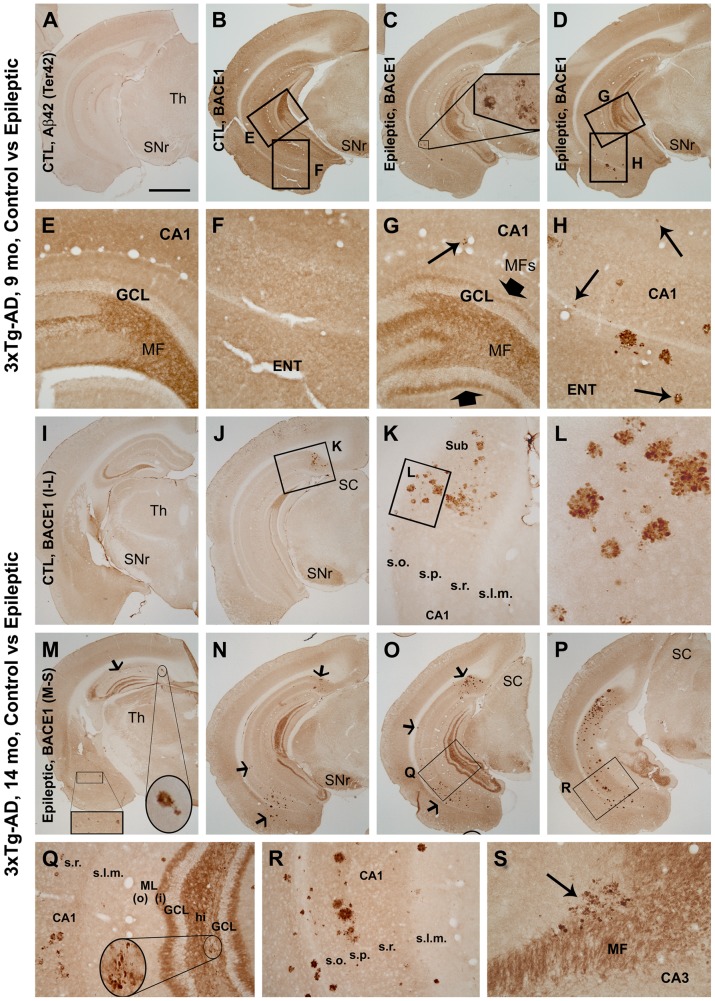
Altered distribution of β-secretase-1 (BACE1) immunoreactivity (IR) in temporal lobe areas of pilocarpine-induced epileptic 3×Tg-AD mice relative to age-matched saline-treated non-epileptic 3×Tg-AD controls. Framed areas in low magnification images are enlarged as indicated to show details of labeling; animal ages and treatment are indicated. Panel **A** shows the absence of amyloid plaques or cellular labeling in the cortex and hippocampal formation in a 9-month old control mouse by the rabbit antibody Ter42, raised against the Aβ C-terminal). Panel **B** and enlargements (**E, F**) show BACE1 IR in the cortex and hippocampal formation in this control mouse, appearing as neuropil labeling except for strong IR in mossy fiber (MF) terminals in the hilus and CA3 (**E**). Panels **C, D** and **G** show BACE1-labeled mossy fiber sprouting (MFs, indicated by large arrows in **G**) in the inner dentate molecular layer in a 9-month old epileptic mouse. BACE1 also labels a small number of plaque-like profiles in the temporal lobe areas (**G, H**, small arrows). These BACE1 labeled elements appeared to be swollen/sprouting neurites, occurred discretely or in clusters of varying sizes, and were seen in both the hippocampus (**H**) and temporal cortex (**C, D, H**). Panels **I–L** show BACE1 IR in a 14-month old control mouse; labeled neurites and neuritic clusters were found in the subiculum in caudal sections (**J–L**). Panels **M–S** show BACE IR in an epileptic 3×Tg-AD mouse at the same age; many more BACE1-labeled neurites and neuritic clusters were throughout the hippocampus as well as in the amygdala and temporal cortex. Some large neuritic clusters are indicated by arrows (**M–O**). Mossy fiber sprouting is clearly seen in the inner molecular layer at low (**M–O**) and high (**Q**) magnifications. A few BACE1 labeled neuritic clusters were seen in the mossy fiber zone in the hilus (**Q**, inset) and CA3 (**S**, arrow). GCL: granule cell layer; SC: superior colliculus; SNr: substantia nigra, pars reticulate; Th: thalamus; ML(i) and ML(o): inner and outer molecular layer; hi: hilus; s.o.: stratum oriens; s.p.: stratum pyramidale; s.r.: stratum radiatum; s.l.m.: stratum lacunosum-moleculare. Scale bar in (A) = 1 mm in (**A**) applying to (**B–D, I, J, M–P**); equivalent to 250 µm for (**E–H, K, Q, R**), 100 µm for (**L, S**).

In 9-month old control 3×Tg-AD mice, no extracellular Aβ immunoreactivity (IR) was observed in the cerebral cortex and hippocampal formation as verified using several different Aβ antibodies (listed in Table 1), including two rabbit antibodies raised against the C-terminus of the Aβ domain, i.e., Ter40 (Aβ36–40; not shown) and Ter42 (Aβ38–42; Fig. 1A), respectively [29]. At this pre-plaque age, the overall distribution pattern of BACE1 IR in the forebrain was largely comparable to that seen in non-transgenic rodents [30]. Specifically, BACE1 IR was present in neuropil of the cortex, amygdala and hippocampal formation, with little IR in cortical white matter. Strong BACE1 labeling was observed in the hippocampal mossy fiber field, but only faint labeling was seen in the pyramidal cell and dentate granule cell layers (Fig. 1B, E and F).

In the 9-month old epileptic 3×Tg-AD mice (3 months after epilepsy induction), the distribution pattern of BACE1 was altered in both the hippocampal formation and other temporal cortical structures (Fig. 1C, D, G and H). A strong band of BACE1 IR was observed along the border of the inner molecular layer and the granule cell layer of the dentate gyrus in epileptic mice, representing aberrant mossy fiber sprouting into this zone (Fig. 1C, D and G). In addition, discrete regions containing heavy BACE1 IR, appearing as swollen/sprouting neurites and neuritic clusters of varying size, were seen in the hippocampal formation, amygdala and temporal cortex in the epileptic animals (Fig. 1C, D and H).

Extracellular plaques were rarely detected in 11-month old control 3×Tg-AD mice from our colony, but were consistently seen in 14 month-old non-epileptic 3×Tg-AD mice (See [29]). In this latter age group, neuritic plaques were often located around the subiculum, especially at caudal/temporal levels of the hippocampal formation. These neuritic plaques consisted of BACE1-labeled dystrophic neurites (Fig. 1J–L) associated with local extracellular Aβ deposits (see also [29]). In contrast to controls, in both 11- (3 months after epilepsy induction; images not shown) and 14-month (6 months after epilepsy induction) old epileptic 3×Tg-AD mice, BACE1 IR was clearly present in the inner molecular layer of the dentate gyrus (Fig. 1M–O, Q). In addition, BACE1-labeled dystrophic neurites and neuritic clusters were detected in the hippocampal formation, amygdala and overlying temporal cortex (Fig. 1M–R). Neuritic clusters could occasionally be found in the region of the mossy fiber pathway in the hilus and CA3 in the epileptic animals (Fig. 1Q, S). The overall morphology of BACE1-labeled dystrophic neurites and clusters was comparable between epileptic and non-epileptic transgenics (Fig. 1K, L, Q–S).

**Figure 2:**
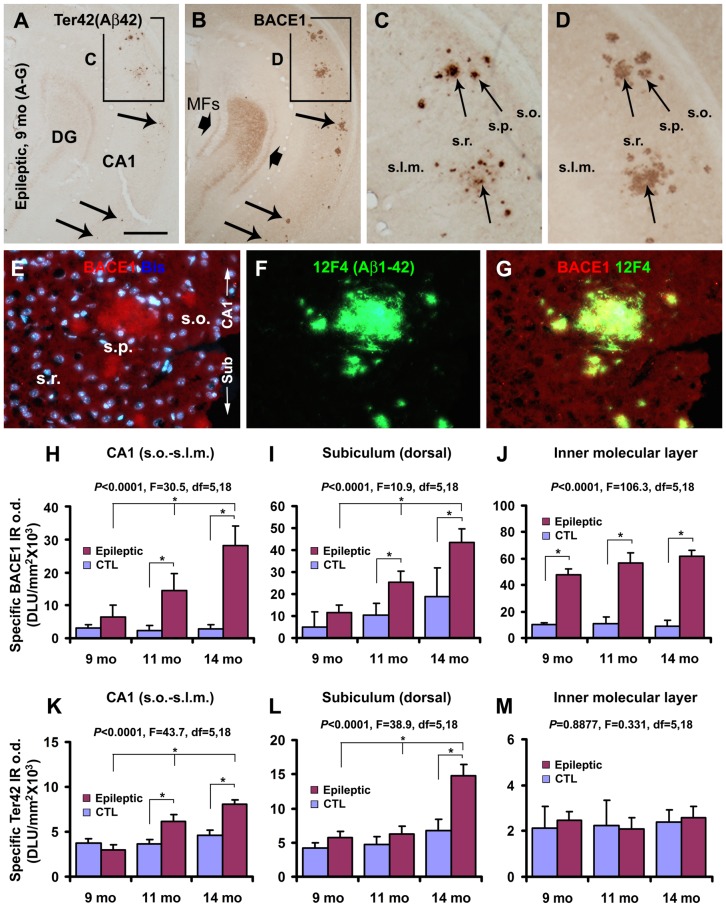
Representative images from a 9-month old epileptic 3×Tg-AD mouse showing colocalization of BACE1-labeled dystrophic neurites with local extracellular Aβ deposits. The colocalization of Ter42/12F4 and BACE1 IR is illustrated between consecutive sections (**A–D**) and in double immunofluorescence (**E–G**). In some large neuritic plaques, the amyloid core (with heavy Aβ IR, arrows) corresponded to a central zone of the neuritic cluster with reduced BACE1 IR (**C, D**). Note the colocalization of BACE1 IR and 12F4 IR around small as well as large neuritic plaques (**E–G**). Bar graphs (**H–M**) quantify the density of BACE1 and Ter42 labeling in temporal regions of epileptic vs control 3×Tg-AD mice. The specific optical density (o.d.) of BACE1 IR measured in CA1 did not significantly change in vehicle-treated mice, but increased from 9 to 14 months of age in the epileptics (**H**). In the subiculum (**I**), specific BACE1 o.d. tended to increase from 9 to 14 months of age in both groups, but significantly in the epileptics. Specific BACE1 o.d. in the inner dentate molecular layer did not increase with age in the epileptic mice, but was dramatically higher than controls at all three age points (**J**). Specific o.d. for Ter42 IR increased in CA1 (**K**) and subiculum (**L**) with age in the epileptic mice. No age- or group-related differences in Ter42 IR were found in the inner molecular layer (**M**). Abbreviations are as defined in Figure 1; large arrows bracket mossy fiber sprouting (MFs) in **B**. Scale bar in (**A**) = 250 µm for (**A, B**), 100 µm for (**C, D**) and 50 µm for (**E–G**).

Localized extracellular Aβ deposition was detected with the Ter40 (not shown) and Ter42 antibodies in temporal lobe structures of 9–14 month old epileptic, and 14-month old control, 3×Tg mice. These deposits appeared to co-exist regionally with dystrophic neurites that exhibited increased BACE1 IR (Fig. 2A–D). Using double immunofluorescence and the monoclonal Aβ antibody 12F4 that detects only extracellular Aβ deposits [39],[40], BACE1 labeled neurites were locally associated with extracellular Aβ aggregates in the hippocampal formation and extra-hippocampal limbic structures in both control and epileptic transgenic mice (Fig. 2E–G), arranged as the so-called primitive and cored neuritic plaques [30],[41].

Densitometric analyses confirmed that there was an increase (sometimes age-related) in plaque-associated BACE1 IR (Fig. 2H–J) and Ter42 IR (Fig. 2K–L) in the hippocampal formation of epileptic 3×Tg-AD mice relative to controls. Thus, the mean specific o.d. of BACE1 IR in CA1 progressively increased from 9 to 14 month of age in the epileptic mice (*p*<0.0001, one-way ANOVA). Bonferroni multiple comparison tests revealed statistically significant differences (*p*<0.05 to *p*<0.001) between the epileptic and control mice at 11 and 14 months of age (Fig. 2H). Specific BACE1 o.d. in the dorsal subiculum tended to increase from 9–14 months in the epileptic and control groups (*p*<0.0001, one-way ANOVA), more robust in epileptic relative to control groups at 11 and 14 months of age (Fig. 2I). Reflecting mossy fiber sprouting, specific o.d. of BACE1 IR was increased in the inner molecular layer of the dentate gyrus in epileptic relative to control 3×Tg-AD mice (*p*<0.0001, one-way ANOVA) at all three ages examined (Fig. 2J).

Specific o.d. of Ter42 IR increased in CA1 from 9–14 months of age in epileptic 3×Tg mice (*p*<0.0001, one-way ANOVA), but was significantly higher than that in non-epileptic controls at 11 and 14 months (Fig. 2K). Increased o.d. of Ter42 labeling was quantitatively confirmed in the subiculum of epileptic 3×Tg mice relative to controls at 14 months of age (Fig. 2L). In contrast, no age or group-related differences existed for the o.d. of Ter42 labeling in the inner molecular layer of the dentate gyrus (Fig. 2M).

**Figure 3:**
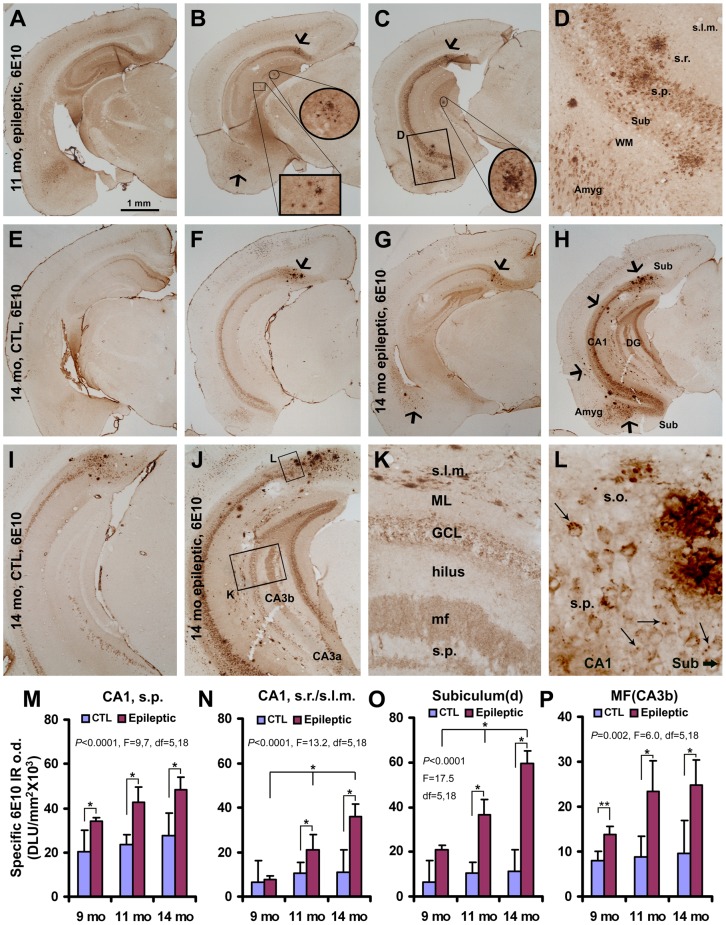
Neuronal and extracellular labeling with 6E10 antibody in the cortex and hippocampal formation of epileptic and control 3×Tg-AD mice at indicated ages (A–L). 6E10 IR was present in pyramidal neuron somata in deep cortical layers, amygdala, subiculum, CA1 and CA3a in normal and epileptic 3×Tg-AD mice. The antibody also visualized extracellular plaques in the subiculum, hippocampus, dentate gyrus, temporal/pyramidal cortex and amygdala in 11-monthold epileptic transgenics (**B–D**), but not in controls at this age (images not shown). In 14- month old controls, 6E10 labeled plaques were seen in the subiculum in caudal sections (**F**, arrow; shown at higher magnification in **I**); no plaques were seen in the hippocampal proper (**E, F**). In contrast, in 14-month old epileptic transgenics, plaques occurred in the subiculum, CA subfields and dentate gyrus as well as in temporal lobe cortex and amygdala (examples indicated by large arrows in **G, H**; **H** is shown at higher magnification in **J–L**). At higher magnification, some 6E10-labeled swollen processes were found in stratum lacunosum-moleculare (s.l.m.; **K**) and labeled perisomatic elements were seen around subicular and hippocampal pyramidal neurons (**L**, arrows). 6E10 IR was also elevated in the somata of cortical and hippocampal pyramidal neurons and the dentate granule cells in epileptics (**H, J**) relative to controls (**F, I**). Bar graphs (**M–P**) compare optical density measurements in selected hippocampal subregions in 9-, 11- and 14-month old mice from both groups. Quantitatively, 6E10 IR in the epileptic mice progressively increased in stratum pyramidale (s.p.; **M**) and stratum radiatum (s.r.)/lacunosum-moleculare (s.l.m.) of area CA1 (**N**), and in the dorsal subiculum (**O**) from 9–14 months in the epileptic mice. In addition, specific 6E10 o.d. in CA3b mossy fiber terminals (mf) in epileptics increased from 9–14 months of age and was higher relative to non-epileptic controls at each age point. Scale bar in (**A**) = 1 mm in (**A**) applying to (**B, C, E–H**); equivalent to 500 µm for (**I, J**), 200 µm for (**D, K**), and 100 µm for (**L**).

The monoclonal Aβ antibody 6E10 labels mutant human APP in neuronal somata in addition to extracellular Aβ aggregates [29],[40],[42]. We analyzed the distribution and density of 6E10 IR in epileptic relative to control 3×Tg-AD mice. Small amounts of 6E10-labeled extracellular Aβ plaques occurred in the hippocampal formation, amygdala and temporal lobe cortex in 11-month old epileptic (Fig. 3A–D), but not in age-matched control (images not shown), 3×Tg-AD mice. By 14 months of age, 6E10-labeled extracellular deposits were detectable in control brains in the subiculum at caudal hippocampal levels (Fig. 3F, I). In contrast, in 14 month old epileptic 3×Tg-AD mice, increased extracellular 6E10 IR was present throughout the hippocampal formation and temporal cortex (Fig. 3G, H, J–L). 6E10 IR in the hippocampal pyramidal and the dentate granule cell layers also appeared stronger in epileptic relative to control mice (Fig. 3J compared to 3I). 6E10 IR revealed the mossy fiber terminals in epileptic mice (Fig. 3J, K), whereas little to no IR was observed in this location in control mice (Fig. 3I). At higher magnification, 6E10 IR appeared to be associated with swollen processes in stratum oriens (Fig. 3L) and stratum lacunosum-moleculare (Fig. 3K), and punctuate terminal-like endings around the somata of some pyramidal neurons (Fig. 3L).

Quantitatively, the specific o.d. of 6E10 IR in CA1 stratum pyramidale was significantly increased in epileptic relative to control 3×Tg-AD mice at 9, 11 and 14 months of age (*p*<0.0001, one-way ANOVA with Bonferroni's multiple comparison test; Fig. 3M). Specific 6E10 o.d. in the stratum oriens (images not shown) and the apical dendritic regions (Fig. 3N) of the CA1 pyramidal neurons was increased with age in epileptic 3×Tg-AD mice (*p*<0.0001, one-way ANOVA), greater than that for the corresponding control groups at 11 and 14 months of age. Similarly, an age-related increase in o.d. for 6E10 IR in the epileptic mice was found in the dorsal subiculum (*p*<0.0001, one-way ANOVA; Fig. 3O) and in the CA3b mossy fiber terminal field of epileptic 3×Tg-AD mice relative to controls (*p* = 0.002, one-way ANOVA; Fig. 3P).

**Figure 4:**
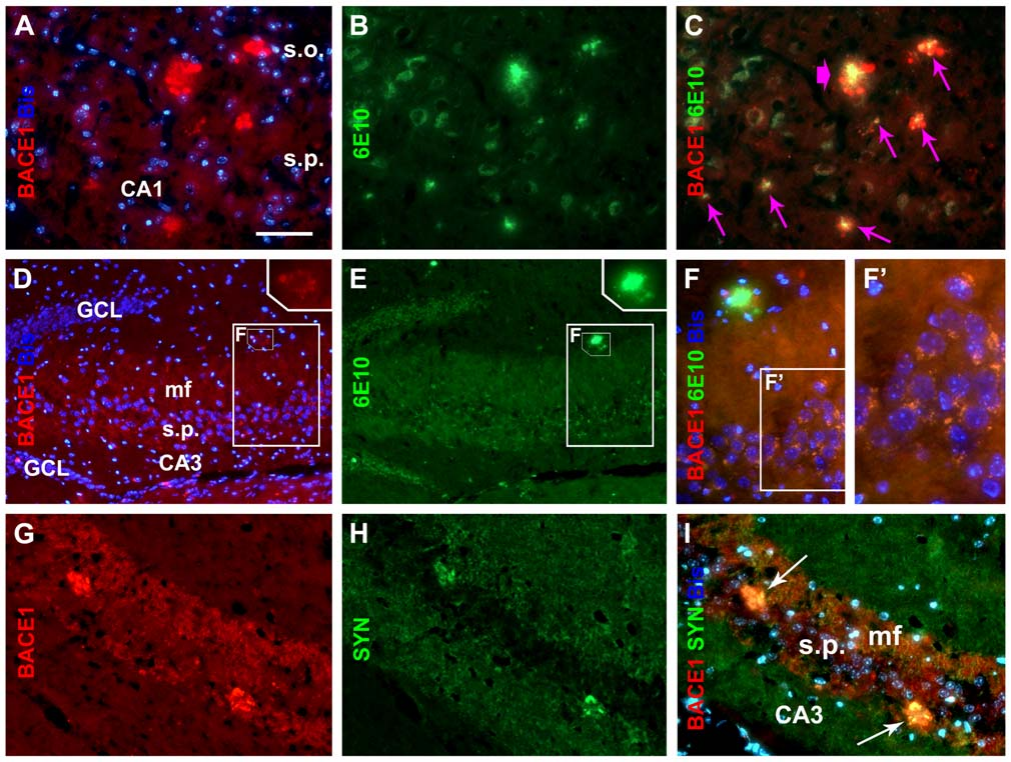
Double immunofluorescence illustrating the relationship of BACE1 IR to 6E10 and synaptophysin-IR in a hippocampal section from a 9-month old epileptic 3×Tg-AD mouse. Bisbenzimide (blue fluorescence) nuclear counterstain is included in some image panels. Top panels (**A** & **B**; merged in **C**) show several small (small arrows in **C**), and one large cored (large arrow in **C**), neuritic plaques expressing 6E10 IR surrounded by BACE1 IR. Middle panels (**D** & **E**; merged in **F**) show localization of BACE1 and 6E10 IR in mossy fiber terminals. A small neuritic plaque is present in the field, indicated in the highlighted rectangle and enlarged in the upper right of **D** and **E**, again shows BACE1-labeled swollen neurites surrounding bright 6E10 extracellular IR. Granule-like BACE1 profiles are present around CA3 pyramidal neurons (**F, F′**). Lower panels (**G** & **H**; merged in **I**) show two heavily BACE1-reactive neuritic clusters, located in CA3 pyramidal cell layer, that colocalize with synaptophysin (SYN). Other abbreviations are defined in the legend of Fig. 1. Scale bar = 100 µm in **A** applying to (**B–E**, **G–L**), equal to 50 µm for (**F**) and 25 µm for (**F′**).

Focal colocalization of dystrophic neurites with extracellular Aβ deposits was evaluated with BACE1/6E10 double immunofluorescence in epileptic transgenic mice. BACE1 and 6E10 IR were visualized in both primitive and cored neuritic plaques of varying sizes (Fig. 4A–C; examples are from a 9-month old mouse). In addition, increased non-cellular 6E10 IR was localized in the mossy fiber terminal zone in CA3 that concurrently expressed strong BACE1 IR (Fig. 4D–F). Further, double-labeled punctuate profiles were sometimes seen in CA3 pyramidal cell layer (Fig. 4F, F′). The focal increase in BACE1 IR colocalized with synaptophysin IR, including in and around the mossy fiber field (Fig. 4G–I), consistent with our earlier reports [29],[30],[31].

### Altered p-tau labeling in epileptic 3×Tg-AD-AD mice

**Figure 5:**
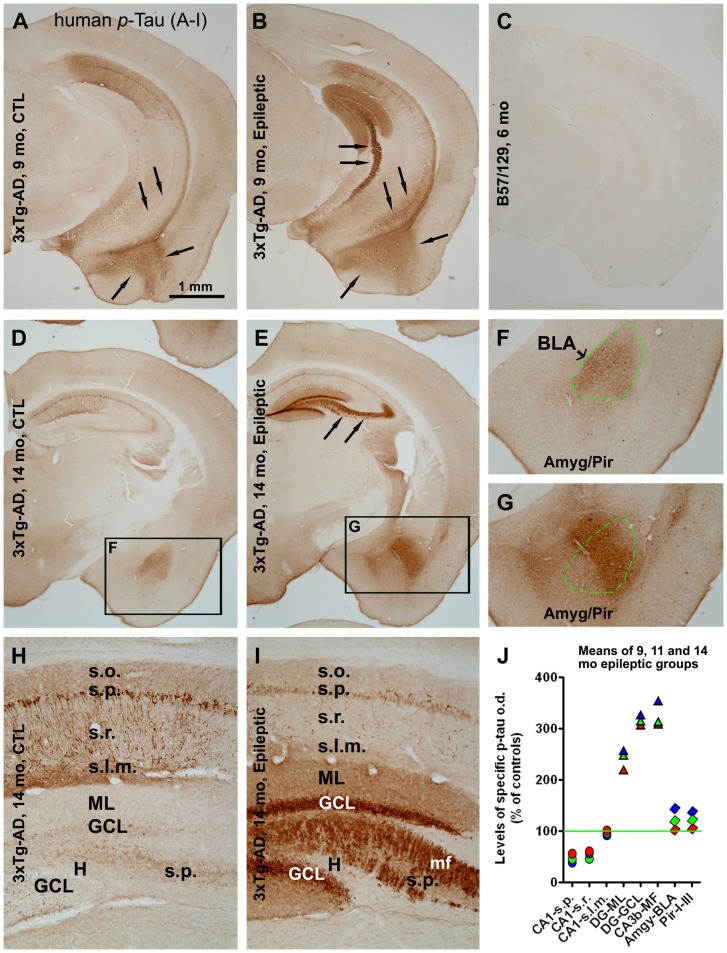
Representative images and densitometry showing altered phosphorylated-tau (p-tau) expression in epileptic relative to control 3×Tg-AD mice. Top panels illustrate *p*-tau IR in the hippocampus of 9 month old (**A** vs **B)** and 14 month old (**D** vs **E**) epileptic animals. Enhanced p-tau IR was observed in the dentate gyrus and mossy fiber zone (arrows in **B** and **E**). No p-tau IR was seen in the brains of wild-type mice of the same background strain as the transgenics (**C**). Insets in **F** and **G** show enhanced p-tau IR in the basal lateral nucleus of the amygdala (BLA, outlined by green dashed line) and the piriform cortex. Higher power images (**H, I**) of the hippocampus of 14 month old animals further illustrate enhanced p-tau IR in the dentate gyrus, including the molecular layer (ML), granular cell layer (GCL) and mossy fibers (MF), but reduced p-tau IR in the hippocampus CA1 (all strata), in the epileptic relative to non-epileptic 3×Tg mice. Graph (**J**) summarizes densitometric data for p-tau IR measured over limbic regions as indicated. The green line on the graph represents the level of mean density (defined as 100%) from all control animals (n = 12), used to normalize specific densities in corresponding areas of interest in individual animals. Each symbol (circle, triangle or diamond) in the graph represents the normalized mean density for a given lamina/area in one age group of epileptic mice (9 month: red; 11-month: green; 14-month: blue). The quantitative data reinforce that p-tau IR was reduced in CA1, but markedly increased in the dentate gyrus, of epileptic mice. Abbreviations are as defined in Fig. 1. Scale bar in (**A**) = 1 mm in (**A**) applying to (**B–E**); equivalent to 400 µm for (**F, G**), and 200 µm for (**H, I**).

P-Tau IR was assessed in the cortex and hippocampal formation with rabbit and mouse antibodies against hyperphosphorylated human tau (detailed in Table 1). As an initial control we determined that no p-Tau IR was observed in the forebrain of non-transgenic mice (Fig. 5C). Overall, the pattern of p-Tau IR was different in the forebrain of epileptic 3×Tg mice (Fig. 5B, E, G and I) compared to non-epileptic controls (5A, D F and H). p-Tau IR was heavily expressed in the dentate gyrus of epileptic mice, including in the molecular layer, granule cell layer and mossy fiber terminal zone (Fig. 5B, E and I). In contrast, p-Tau IR in epileptics was reduced relative to controls in the hippocampus proper, especially in CA1 (Compare Fig. 5I to 5H.) p-Tau IR in the neuropil appeared to be increased in the amygdala, and to a lesser extent in the piriform cortex, in epileptic 3×Tg-AD mice relative to controls (Fig. 5D–G). Tangled neurons and neurites were occasionally seen in areas that showed an overall increase of p-tau IR (Fig. 4I).

Quantitatively (Fig. 5J), p-tau IR in the epileptic mice was significantly reduced in CA1 stratum pyramidale (s.p.) to 53.4±4.9% (mean ± s.e.m.), 48.2±6.0% and 38.4±4.8% at 9, 11 and 14 months of age relative to controls (100±2.6%; n = 12; p = 0.005, F = 25.6, df = 3, 20); the differences between epileptics and controls were significant at all age points. The density of p-tau IR density in CA1 stratum radiatum was 56.6±4.4%, 42.4±3.5% and 50.4±3.5% in the epileptic mice at 9-, 11- and 14-month old epileptic mice relative to controls (100±2.1%; p = 0.011, F = 15.9, df = 3, 20). In the dentate gyrus, p-tau IR was significantly increased in all subregions at all ages: (1) in the molecular layer to 221.0±34.0%, 246.4±53.0% and 258.2±50.1% of controls (100±12.9%; p<0.0001, F = 38.8, df = 3, 20); (2) in the granule cell layer to 309.2±39.8%, 319.1±44.5% and 330.4±40.8% of controls (100±9.9%; p<0.0001, F = 115.5, df = 3, 20); and (3) in mossy fiber zone (CA3a) to 310.6±53.7%, 314.9±61.4% and 357.6±66.1% of controls (100±13.9%; p<0.0001, F = 59.9, df = 3, 20).

The density of p-tau IR in the basal lateral nucleus of the amygdala (BLA) of 9-, 11- and 14-month old epileptic mice showed a progressive increase, i.e., 110.8±15.3% (n.s.), 125.2±33.5% (n.s.) and 142.5±16.7% (p<0.01) of control levels (100±3.9%; overall p<0.001, F = 8.02, df = 3, 20). Similar changes in p-Tau IR were seen in the piriform cortex of the epileptic mice: 109.5±14.9% (n.s.), 117.0±19.3% (n.s.) and 127.9±26.1% (p<0.05) of control levels (100±1.4%; overall p = 0.013, F = 4.85, df = 3, 20).

### Neuronal loss in epileptic 3×Tg-AD mice

**Figure 6:**
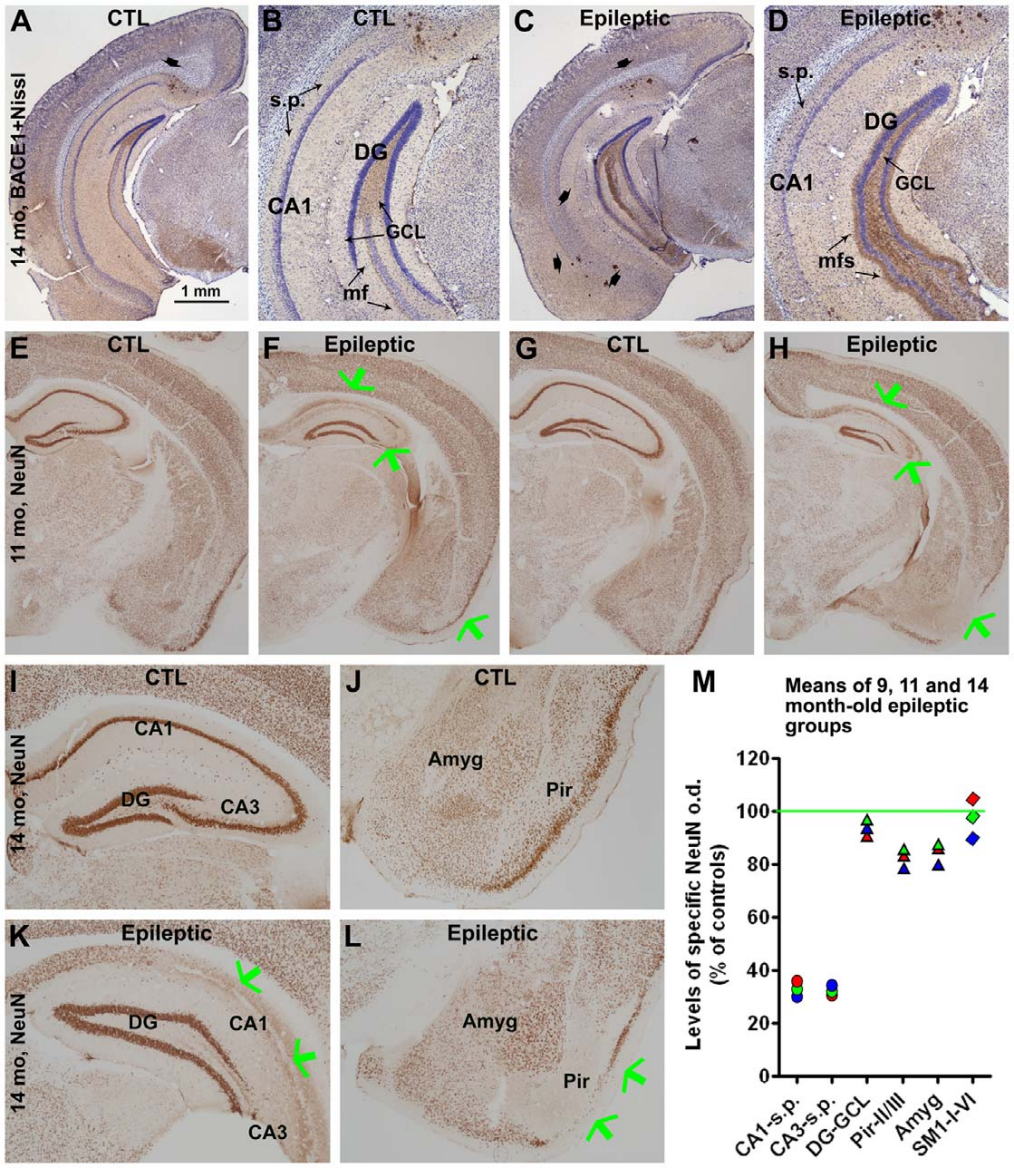
Images and densitometry showing cell (A–D) and neuronal (E–M) loss in temporal lobe areas in epileptic relative to control 3×Tg-AD mice. Panels (**A**–**D**) shows BACE1 immunolabeled sections, counterstained with cresyl violet, that reveal cell loss in CA1 and CA3 stratum pyramidale (s.p.) in a 14 month-old epileptic mouse (**C**, **D**) relative to an age-matched control (**A**, **B**). Arrows in **A** and **C** point to relatively large neuritic clusters. Mossy fiber sprouting (mfs) in the inner molecular layer is clear in **D**. Profound loss (green arrows) of immunoreactivity for neuron-specific nuclear antigen (NeuN IR) can be seen in CA1 and CA3, and to a lesser extent in the piriform cortex and amygdala in 11- (**F**, **H**) and 14- (**K**, **L**) month old epileptic mice relative to controls (**E, G, I, J**). Graph (**M**) summarizes normalized levels of densitometric data of NeuN IR in the hippocampal cell layers, amygdala and piriform cortex. The green line represents the mean density (defined as 100%) from all control animals (n = 12). Symbols (circle, triangle or diamond) in the graph represent normalized means from the 3 age groups of the epileptics (9-month: red; 11-month: green; 14-month: blue). NeuN density in somatosensory cortex is shown as negative assay control. Refer to Fig. 1 for abbreviations. Scale bar in (**A**) = 1 mm in (**A, C, E–H**); equivalent to 250 µm for (**B, D, I–L**).

Regional cell loss was readily detectable in epileptic relative to control 3×Tg-AD mice in BACE1-labeled sections counterstained with cresyl violet (Fig. 6A–D). NeuN immunolabeling was carried out to assess the pattern and extent of neuronal loss in epileptic versus control animals. Relative to control 3×Tg mice, reductions in NeuN IR were seen in the temporal cortex, amygdala and hippocampus proper in epileptic 3×Tg-AD mice (Fig. 6F, H, K and L). Quantitatively (Fig. 6M), NeuN IR in CA1 stratum pyramidale was significantly reduced in epileptics at all ages examined (9 months: 35.4±7.1%, p<0.001; 11 months: 33.1±8.0%, p<0.001; 14 months: 30.4±6.8%, p<0.001) relative to controls (100±1.5%, n = 12; p<0.0001, F = 354.9, df = 3, 20). A comparable reduction was seen in the CA3 pyramidal cell layer (Fig. 6M). In the BLA, NeuN IR declined to 86.9±6.2% (p<0.001), 87.0±7.8% (p<0.001) and 79.1±5.2% (p<0.001) in 9-, 11- and 14-month old epileptic mice relative controls (100±1.9%; p<0.0001, F = 26.0, df = 3, 20). Further, NeuN IR in piriform cortical layers II/III in 9, 11 and 14 month-old epileptic mice was reduced to 83.9±13.0% (p<0.01), 86.5±7.0% (p<0.05) and 77.0±5.7% (p<0.001) of control levels (100±4.7%; p<0.0001, F = 13.6, df = 3, 20). No significant group differences were found in NeuN IR in dentate granule cell layer or the somatosensory cortex. In all regions where neuron loss was found, the effect already appeared to be maximal at 9 months of age, the youngest group examined.

## Discussion

The pilocarpine model of epilepsy recapitulates major pathological characteristics of human TLE. However, pilocarpine administration does not always induce acute status epilepticus that results in chronic epilepsy in rodents. Spontaneous recurrent convulsant seizures, together with pathological findings of hippocampal mossy fiber sprouting and neuronal death, are commonly used to confirm the establishment of chronic temporal lobe epilepsy in this rodent model [34], [35], [36]. Since 3×Tg-AD mice develop both plaques and tangles [33], they provide an excellent model system for investigating the interaction of chronic epilepsy with the development of AD-type neuropathology. In the present study, pilocarpine treated 3×Tg-AD mice that developed behavioral and pathological features of chronic TLE were found to exhibit profound changes in AD-like neuropathology relative to controls. Thus, BACE1 IR was increased in the hippocampal formation and temporal cortex, with a new band of supragranular labeling appearing in the dentate gyrus. Extracellular β-amyloid deposition (revealed by 6E10 and Ter42) in temporal lobe structures also developed earlier, and more robustly, in epileptic transgenic mice. Further, p-tau expression was apparently altered in the hippocampal formation in epileptic transgenics relative to controls. Finally, as expected, neuron loss was evident in the hippocampal formation and in some extrahippocampal temporal lobe regions in epileptic 3×Tg-AD mice.

### Epileptic 3×Tg-AD mice exhibit increased amyloidogenic protein expression and accelerated plaque pathogenesis in temporal lobe structures

The present study clearly showed that epileptic 3×Tg-AD mice exhibited region-specific increases of BACE1 expression, relative to controls, that was largely associated with aberrant axonal sprouting and axonal pathology. Mossy fiber sprouting into the inner molecular layer was distinctly labeled by the BACE1 antibody in epileptic mice surviving 3–6 months after pilocarpine-induced status epilepticus, recapitulating the characteristic pathological pattern of aberrant axonal sprouting in TLE [2],[6],[21],[34],[35]. In addition, BACE1-labeled sprouting/swollen axonal neurites and neuritic clusters appeared earlier and more widely in epileptics relative to controls. This localized axonal pathology occurred in temporal lobe structures including the subiculum, amygdala and piriform cortex in addition to the hippocampus and dentate gyrus. The BACE1-labeled sprouting/swollen axonal profiles were site-specifically associated with local extracellular Aβ deposition as verified by mirror section comparison and double immunofluorescence. Importantly, this experimentally-induced BACE1 and extracellular Aβ IR emerged several months earlier than the usual age for Aβ plaque development in this mouse line [29],[40]. These results suggest that pathological axonal sprouting and swelling inherent with BACE1 elevation might lead to accelerated neuritic plaque formation in epileptic temporal lobe structures [29,],[30],[31],[32].

Previous studies have shown that neuronal activity enhances Aβ release from synaptic terminals and elevates interstitial fluid Aβ levels [43],[44], providing a foundation for the suggestion that increased amyloid pathogenesis may occur as a result of neuronal hyperactivity [23],[24]. However, it should be noted that, although localized neuronal hyperactivity occurs during an epileptic episode, chronic temporal lobe epilepsy is characterized by neuronal *hypo*metabolism during the interictal period, the more sustained pathophysiological and clinical state of the disease [10],[11],[12],[13]. Recent studies indicate that physiological activity inversely regulates BACE1 messenger and protein expression in neurons, including at axonal terminals [45],[46],[47]. Thus, increased amyloidogenesis might occur as a result of BACE1 upregulation due to cerebral hypometabolism in chronic TLE. Alternatively, the elevated BACE1 expression might occur as a part of intrinsic modulation of altered synaptic/axonal plasticity, a pathological hallmark of temporal lobe epilepsy [45],[48],[49],[50].

Increased neuronal expression of APP has been found in samples of epileptic human temporal lobe cortex [51]. In the present study, increased 6E10 IR was found in epileptic, relative to control, neuronal somata and axonal terminals in temporal lobe regions. The former change was mostly evident in the hippocampal pyramidal cell and dentate granule cell layers, while the latter was clearly present in CA3 mossy fiber terminals. The increased intrasomal 6E10 labeling in the epileptic 3×TgAD brains likely represents augmented intraneuronal expression of transgenic human APP [29],[42]. The elevated 6E10 IR in mossy fiber terminals of epileptic mice might also reflect elevated intra-axonal APP β-site cleavage fragments (β-CTF) [29], given the increased BACE1 expression at this same location.

One intriguing finding of this study is that, although the aberrantly sprouted mossy fiber terminals exhibited fairly heavy BACE1 IR in the dentate inner molecular layer and in CA3, extracellular Aβ IR occurred only locally within the mossy fiber field, restricted to loci with morphologically swollen neurites. It is possible that sprouting mossy terminals release an increased amount of soluble Aβ into local extracellular space, but at a level that does not reach the threshold for fibrillation and aggregation. Alternatively, synaptic structural integrity may be a key factor determining local Aβ aggregation/deposition. In this scenario presynaptic terminals, if they do not “properly” form synapses with post-synaptic counterparts, may be prone to dystrophic pathogenesis (e.g., sprouting/swelling) that would lead to continuous Aβ production and release, eventually resulting in local Aβ accumulation and deposition (i.e, the formation of neuritic plaques). This speculation is consistent with electron microscopic studies indicating that plaque-associated dystrophic axonal terminals are often not partnered with postsynaptic components [41]. However, the majority of sprouted mossy terminals in epileptic hippocampal area CA3 and in the dentate gyrus seem to have intact synaptic structural integrity, forming synapses capable of, including abnormal, neurotransmission [52],[53],[54]. Future comparative electron microscopic characterization of small and large BACE1 labeled sprouting/swelling neurites might resolve this issue, as well as revealing the earliest dystrophic changes at synaptic/axonal terminals which trigger the formation of neuritic plaques.

### Epileptic 3×Tg-AD mice show differential p-tau expression in temporal lobe structures

Intraneuronal tangle formation has been reported in the dentate gyrus of humans with TLE [17] or epileptic cortical dysplasia [18]. Other studies have reported p-tau expression in mossy fiber terminals in rat models of epilepsy [20],[21]. In the present study, differential changes in p-tau labeling are found in epileptic 3×Tg-AD mouse temporal lobe areas as revealed by two antibodies that specifically label human p-tau. There is a significant reduction of p-tau IR in hippocampal CA1, likely due to the loss of pyramidal neurons in this region (see the next section). In contrast, p-tau IR is strikingly increased in the dentate gyrus and noticeably in the amygdala and piriform cortex. Very strong p-tau IR labeling appears in the molecular layer, granule cell layer and mossy fiber terminals in the epileptic mice. Unlike the amyloid pathology, there are no significant age-related alterations in p-tau IR, probably because the modulation exhibits a “ceiling effect”.

In normal adult brain tau protein is largely present in axons, bound to tubulin to provide microtubule stabilization, possibly with a low rate of phosphorylation to allow cytoskeleton flexibility and neuroplasticity [55],[56]. p-Tau expression occurs normally during brain development [57]. In AD and other neurodegenerative disorders, tau may be hyper-phosphorylated and disassociated from microtubules, resulting in p-tau aggregation and the formation of neurofibrillary tangles in neuronal somata and dendrites [58],[59]. Dentate granule cells are among the most plastic neurons in the brain since they are renewed via adult neurogenesis [60]. It was recently shown that a tau protein isoform is a marker for new granule cells and their axons [61]. Thus, p-tau up-regulation in these neurons might be related to altered neuroplasticity, including axonal reorganization in the epileptic brain.

### Neuronal loss coexists regionally with amyloidogenic and p-tau alteration in 3×Tg-AD mice

Neuronal death is a well-documented phenomenon in the pilocarpine rodent model of epilepsy. Degenerating neurons can be labeled with Fluoro-Jade C or Dark Neuron stain hours after pilocarpine-induced seizures [62],[63]. Hippocampal pyramidal neurons in CA3 and CA1, as well as interneurons in the hippocampus and dentate gyrus, are lost in epileptic animals [2],[6],[9]. Neuronal damage and death also occur in other limbic areas, including the amygdala and temporal cortex [7],[64],[65],[66]. Both excitotoxic and ischemic mechanisms may be involved in early neuronal damage and death in the pilocarpine model of epilepsy [67].

Consistent with the above, cell loss is clearly observed in the present study in Nissl-stained sections through hippocampal areas CA1 and CA3 in epileptic 3×Tg-AD mice relative to controls. Neuronal loss is confirmed by the finding of reduced NeuN IR the CA1 and CA3 pyramidal cell layers, and to a lesser extent, in piriform cortex and amygdala. Thus, neuronal damage and death regionally coexist with the increased amyloidogenesis and p-tau overexpression in epileptic 3×Tg-AD mice. Given that neuronal damage and death are common factors that can induce reactive axonal sprouting and aberrant plasticity or pathology in many experimental and disease conditions [68],[69],[70],[71], it is possible that cell death may serve as an initial trigger for axonal sprouting, thus intensifying the AD-type pathogenesis in the epileptic brains.

In summary, the present study shows that 3×Tg-AD mice with pilocarpine-induced chronic recurrent epilepsy at a pre-plaque age exhibit accelerated Aβ plaque pathogenesis, increased intraneuronal p-tau expression and neuronal loss in some temporal lobe regions. The increased extracellular Aβ deposition occurs in spatiotemporal association with aberrantly sprouting/swelling axonal neurites that heavily express BACE1. The results suggest that BACE1 (and APP) elevation, as well p-tau overexpression, could be potentially linked to aberrant synaptic/axonal plasticity in experimental epilepsy. Taken together, these data identify a tangible cellular mechanism that may reconcile the comorbidity of AD-type pathologies, plaques and tangles, in chronic temporal lobe epilepsy.
